# The Influence of Mayenite Employed as a Functional Component on Hydration Properties of Ordinary Portland Cement

**DOI:** 10.3390/ma11101958

**Published:** 2018-10-12

**Authors:** Zhen He, Yang Li

**Affiliations:** 1 Shaanxi Key Laboratory of Safety and Durability of Concrete Structures, Xijing University, Xian 710123, China; 2 State Key Laboratory of Water Resources and Hydropower Engineering Science, Wuhan University, Wuhan 430072, China; liyangzzu@126.com

**Keywords:** C_12_A_7_, mayenite, function regulator, hydration kinetics process, hydrate assemblage

## Abstract

Influence of C_12_A_7_ (12CaO·7Al_2_O_3_) as a functional component on hydration properties of Ordinary Portland Cement is studied using isothermal microcalorimetric technique, X-ray diffraction analysis, and thermodynamic calculation. Meanwhile, hydrate assemblages are simulated by hydrothermal software. C_2_AH_8_ (2CaO·Al_2_O_3_·8H_2_O) is generated as a transition phase during the hydration of pure C_12_A_7_, while formation of CAH_10_ (CaO·Al_2_O_3_·10H_2_O) is uncertain. Heat-releasing behavior of Ordinary Portland Cement (OPC) could be noticeably affected by C_12_A_7_, especially for the duration of interaction at boundary stage reduces with C_12_A_7_ replacement. Correspondingly, all hydration kinetic parameters first increase and then diminish with C_12_A_7_ replacement. Simulation results manifest in the main hydration products of OPC being ettringite, C-S-H (Calcium-Silicate-Hydrate) gel, portlandite and brucite. Increasing C_12_A_7_ replacement accelerates the consumption rates of gypsum and calcite that are typically included in OPC, and thus the ettringite content is changed and carbonate phases will be produced. Therefore, the microstructure properties of hydrated products of OPC are affected and the compressive strength is influenced. These predications are in good agreement with experimental findings. C_12_A_7_ can be used as a functional component to adjust the consumption rate of suphates in OPC, and also components of carbonate phases can be modified in hydrate assemblage.

## 1. Introduction

Mayenite (12CaO·7Al_2_O_3_ or C_12_A_7_, throughout this article, cement short hand notation is used: A, Al_2_O_3_; F, Fe_2_O_3_; C, CaO; H, H_2_O; C$\bar{C}$, CaCO_3_; S, SiO_2_) is a main mineral phases of calcium aluminate cements (CACs), and will be formed during the formation of C_3_A [1]. Unlike other mineral phases in clinker, C_12_A_7_ possesses the zeolitic structure, and two nearly free bonded O_2_^−^ can be easily substituted by monovalent elements [2,3], such as F^−^, OH^−^, Cl^−^, etc. The derived materials have unique physicochemical properties, and can be used in geopolymeric cements and mineral-based accelerators.

Combining high hydration activity and ion adoption ability of C_12_A_7_, Hirao et al. [4] invented an eco-cement by employing Cl-containing industrial wastes as raw materials, and the cement exhibited a good foreground in solving the industrial pollution. Xie et al. [5] developed a mineral-based sewage purifying agent to eliminate sulphates in waste water, and practical applications proved that the agent could reduce sulphates concentration by at least 75% in 20 min. Moreover, the agent avoided secondary pollution during sewage purification.

However, hydration of C_12_A_7_ could be affected by many factors. Wang et al. [6] reported that increasing initial alkalinity would accelerate the hydration of C_12_A_7_. Han et al. [7] found that rising temperature can also promote the hydration of C_12_A_7_, but it was less effective than increasing initial alkalinity. Edmonds et al. [8] concluded that C_12_A_7_ hydrated fast at 4 °C and very rapidly at 40 °C, whereas the hydration was comparatively low at 20 °C. Damidot et al. [9] reported that hydration of C_12_A_7_ was profoundly boosted by gypsum, due to the fact that the AH_3_ amount generated by the hydration of C_12_A_7_ with gypsum was about two times higher than that of the control group. Park [10] published that the lattice parameters of C_12_A_7_ reduced with F^-^ adoption increasing, also the hydration properties of C_12_A_7_ were affected. 

From the 1980s, C_12_A_7_ have attracted much attention in mineral-based accelerators. Park et al. [11] proved that compared with other silicate- or aluminate-based accelerators, like C_12_A_7_, CaF_2_ and C_4_A_3_s, the demand of C_12_A_7_-based accelerators was least, while the relevant groups had the fastest setting capacity, best mechanical properties, lowest chloride ion permeability, strongest freezing-thawing resistance and smallest strength decrease. Won et al. [12] developed a new type of C_12_A_7_-based accelerator, and the mechanical strength of shotcrete with this accelerator was much high than that of shotcrete with the existing C_12_A_7_-based accelerator. Further, the influence of this new accelerator on the durability of shotcrete was investigated [13], and it was found that shotcrete containing the new accelerator showed higher long-term age strength, better freezing-thawing resistance, lower chloride ion penetration and greater anti-carbonation ability than that of shortcrete with the existing C_12_A_7_-based accelerator. However, it should be emphasized that although the strength of shotcrete including C_12_A_7_-based accelerators could continually increase with hydration age, they were still lower than that of the control groups without C_12_A_7_-based accelerators.

Apart from that, C_12_A_7_ has also been used in geopolymers to improve setting and early-age strength. Rovnanik [14] described that the setting procession of fly ash geopolymer was remarkably promoted by C_12_A_7_, but compared with the control group without C_12_A_7_, early-age strength of groups with C_12_A_7_ increased while long-term strength deteriorated. Majumbar et al. [15] blended C_12_A_7_ with granulated blast furnace slag (GBFS) at a weight ratio of 1:1, and then hydrated the mixtures at various water-to-binder ratios, and cured the samples at different temperatures. Finally, strength reduction was founded at 1 year of age in pure C_12_A_7_ paste, caused by the conversion of aluminate hydrates. However, strength continually increased with hydration age for pastes, mortars and concretes including both C_12_A_7_ and GBFS. The author attributed this phenomenon to the formation of C_2_AsH_8_ (2CaO·Al_2_O_3_·CaSO_4_·8H_2_O) and concluded that C_12_A_7_ can be used in repair work.

Up to now, existing studies on C_12_A_7_ have mainly focused on the heat releasing behavior, setting and hardening properties, mechanical performance and durability, etc. Few reports concerning the influence of C_12_A_7_ on hydration kinetic process and hydrate assemblage of Ordinary Portland Cement (OPC) are published. Therefore, this work primarily investigates these issues.

## 2. Materials and Methods

### 2.1. Raw Materials and Preparing Methods

Pure C_12_A_7_ is synthesized by solid reactions between particles of analytical grade Ca(OH)_2_ and Al(OH)_3_. According to the CaO-Al_2_O_3_ diagram, C_12_A_7_ melts congruently at 1392 ± 5 °C [16]. Formation of the liquid phase can greatly promote reactions between solid particles, but the final products may clot seriously and cause crucible damage. Therefore, calcination temperature is set at 1350 °C in this work. Raw material powders are mixed by the ball grinding method using a high-energy planetary mill of Pulverisette 7 model provided by FRITSCH Company, Markt Einersheim, Germany at 500 revolutions per minute for 10 min a time and repeated three times. Then, the mixed powders are compacted under 30 MPa pressure into cylindrical pellets. Further, these pellets are sintered with a heat rate of 10 °C/min from room temperature to 1350 °C and maintained at the desired temperature for 4 or 8 h in atmosphere. After that, a quenching procedure is conducted by a rapid air cooling method, aiming to maintain the hydration activity of synthesized C_12_A_7_. Free lime (free-CaO) content in synthesized C_12_A_7_ is determined by the glycerine-ethanol method and phase identification is performed by X-ray diffraction (XRD).

In previous studies [11,12,13], it has been pointed out that the optimum dosage of C_12_A_7_-based accelerator locates in the range from 5% to 8%. Though a higher dosage of C_12_A_7_-based accelerator may leads to faster setting and hardening abilities of cement pastes or mortars, their mechanical properties may also be affected. Therefore, in the presented work, C_12_A_7_ substitutes OPC by weight with replacement levels of 2.5%, 5.0% and 7.5%. Both pastes and mortars are prepared to investigate the influence of C_12_A_7_. Pastes with OPC-C_12_A_7_ complex binder hydrate at 20 °C for a certain period and their heat releasing behaviors are monitored by an isothermal calorimeter. The water-to-binder ratio of all paste groups are fixed at 0.4, and samples are labeled as M2, M5 and M7, wherein M signifies that mayenite is involved, and the control group without C_12_A_7_ is labeled as M0. Meanwhile, mortars with OPC-C_12_A_7_ complex binder are also prepared to further examine the influence of C_12_A_7_ on a macro scale. ISO standard sand according to Chinese code is used [17]. The water-to-binder ratio of all mortar groups are fixed at 0.4, and the binder-to-sand ratio is constant at 0.5. The specimen size is 40 mm × 40 mm × 160 mm. Mortars are prepared by an A200C mixer produced by Hobart UK company, Peterborough, Canada. Powders and sands are first stirred slowly in dry state for 60 s, and then water is poured and continuously stirred for another 120 s. After standing for 60 s, the mixture is rapidly stirred for 60 s. The whole mixing procedure lasts for 240 s. Mortars are demoulded in 24 h after pouring, and then cured at 20 °C in Ca(OH)_2_ saturated solution for 14 and 28 d, respectively. Similar to notations of pastes samples, mortar samples are labeled as S0, S2, S5 and S7, wherein the numbers possess the same meaning as that in pastes experiments. Compressive strength and flexture strength of mortars at 14 and 28 d ages are tested. The specified testing procedures can be seen in Chinese code GB/T 17671-1999 [17]. The OPC type is PI 52.5 cement produced by Wuhan Yadong factory, Wuhan, China, complying to Chinese standard GB 175-2007 [18]. The Blaine-specific surface area of selected cement is 341 m^2^/kg. Calculated Gibbs free energy of selected cement is 53.4 kJ/mol, according to the method proposed by Dong [19], wherein Arrhenius Euqation was used. Chemical composition of OPC is compiled in Table 1, and clinker phase contents are calculated based on Bogue equations [20]. As seen in Table 1, silicates are the main mineral phases and account for 70.7% on the whole. Calcite typically included in OPC can be founded. The amounts of gypsum, hemihydrate and anhydrite are determined by thermogravimetric analysis. Generally, readily soluble alkali in OPC are commonly sulphates and their amounts are determined according to ASTM C114-69 through concentrations of easy soluble alkalis in distilled water at a water-to-solid ratio of 10 after an equilibration time of 10 min. The remaining K, Na, Mg and S are assumed to be present as minor constituents in solid solution with the main clinker phases shown in Table 2 [15].

### 2.2. Instruments

Composition of OPC is analyzed by chemical titration and X-Ray Fluorescence (XRF) on Axios provided by PANalytical Company. Thermogravity and differential thermogravimetric (TG/DTG) analysis are used to determine gypsum content on SDT-Q600 model provided by TA Instruments, New Castle, DE, USA, with a resolution of ± 0.0001 mg in temperature range up to 1000 °C. The heating rate is 10 °C /min and the flow of protective gas of nitrogen (N_2_) is 100 mL/min.

Phase identification of C_12_A_7_ and hydrated products are performed via XRD method on Bruker D8 Advanced diffractometer, Karlsruhe, Germany, operating at 40 kV and 30 mA, using Cu Kα radiation and a Ni-filter in 2θ = 10°–70° range. The scanning step is 0.05° and scanning speed is 2 °/min.

Hydration behavior of pure C_12_A_7_ and OPC-C_12_A_7_ complex binder are monitored by isothermal microcalorimetric technique on a TAM Air provided by TA Company. Twenty mililiters of glass ampoules are used and reference material is distilled water. All samples hydrate at 20 °C. Pure C_12_A_7_ sample is mixed by internal stirring for 30 s, while samples with OPC-C_12_A_7_ complex binder are mixed by external stirring methods. The TAM Air maintains a set temperature within ± 0.02 °C and the minimum detection limit is 4 μW.

Hydrate assemblages are simulated by GEMS software, which is a broad-purpose geochemical modeling code that uses Gibbs energy minimization and computes equilibrium phase assemblage in a complex chemical system from its total bulk elemental composition.

### 2.3. Hydration Kinetic Model

Krstulović et al. [21] divided the hydration of Portland cement into three stages, such as the nucleation and crystal growth (*NG*) stage, the interaction at phase boundaries (*I*) stage and the diffusion (*D*) stage. The hydration of *NG* stage can be described by the basic Avrami-Erofeev equation presented as below.

(1) $${\left[-\ln(1-\alpha (t))\right]}^{1/n}=K_{NG}t\;$$

where *t* represents hydration time. α(*t*) signifies the hydration degree at time *t*. *n* denotes reaction order. *K_N_*_G_ means the reaction rate constant of *NG* stage.

The hydration of *I* stage can be described by Equation (2).

(2) $$1-{\left[1-\alpha (t)\right]}^{1/3}=K_{I}t\;$$

where *K_I_* demonstrates the reaction rate constant of *I* stage.

For *D* stage, the following Jander equation is used.

(3) $${\left[1-{\left(1-\alpha (t)\right)}^{1/3}\right]}^{2}=K_{D}t\;$$

where *K_D_* illustrates the reaction rate constant of *D* stage.

Hydration degree is determined through the hydrothermal method according to Equation (4).

(4) $$\alpha (t)=Q(t)/Q_{\infty }\;$$

where *Q*(*t*) is the accumulative hydration heat at time *t*. *Q*_∞_ is the total amount of hydration heat when hydration completely finishes. *Q*_∞_ is calculated by integration over time of the normalized measured heat flow with the correct conversion to J/g.

Calculation methods of the above-mentioned Equations (1)–(3) have been introduced in detail in a previous study [22]. Studies performed by He et al. [23,24], Han et al. [7], Zhou et al. [25] and Tian et al. [26] all confirmed that the Krstulovic-Dabic hydration kinetic model was effective to simulate the hydration process of OPC, sulphoaluminate cement (SAC), and also complex cementitious materials involving silica fume, fly ash or GBFS.

## 3. Results

### 3.1. Hydration Properties of Pure C_12_A_7_

**Figure 1:**
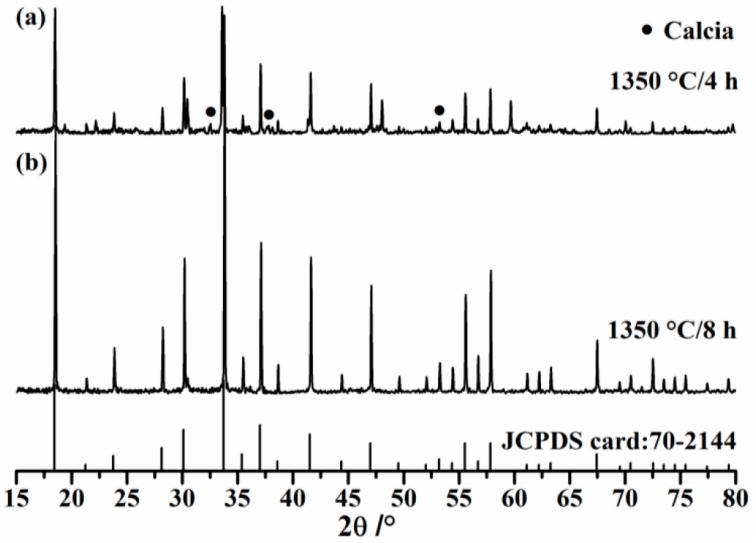
XRD spectra of synthesized C_12_A_7_.

The effect of calcination duration on synthesis effect in C_12_A_7_ preparation is demonstrated in Figure 1. XRD spectra of samples calcined at 1350 °C for 4 and 8 h are shown in Figure 1a,b, respectively. It can be clearly observed that characteristics belonging to calcia (JCPDS file:77-2376) are present in Figure 1a, indicating that free-CaO content is pretty high. However, signals of calcia completely disappear and peak intensities of mayenite increase dramatically in Figure 1b, meaning that C_12_A_7_ (JCPDS file:70-2144) is the only crystal phase. Actually, free-CaO content of synthesized C_12_A_7_ is so low that the solution does not change to red during the refluxing procession, also meaning that free-CaO content of synthesized C_12_A_7_ is under the detection limit of the glycerin-ethanol titration method.

**Figure 2:**
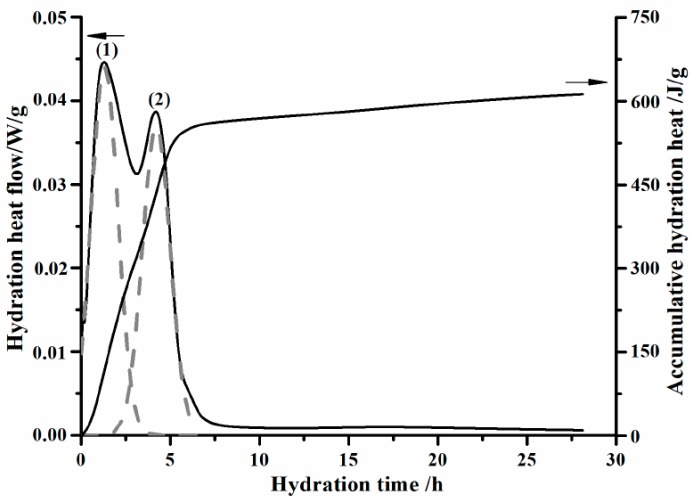
Hydration heat flow and accumulative hydration heat curves of synthesized C_12_A_7_ over hydration time.

Hydration heat flow and accumulative hydration heat during pure C_12_A_7_ hydrating are shown in Figure 2. Pure C_12_A_7_ hydrates at 20 °C with a water to C_12_A_7_ ratio by weight of 10. Two exothermic peaks will appear during the initial 6 h hydration of C_12_A_7_. Appearance times of peak (1) and peak (2) are 1.3 and 4.2 h, respectively. Generally, it is widely accepted that peak (1) correlates to the formation of transition phases, while peak (2) is caused by the conversion of the metastable transition phase to stable hydrogarnet phase of katoite (C_3_AH_6_) [8,9,27]. Actually, the conversion is usually considered as one of the fundamental reasons of volume shrinkage and strength reduction of CACs.

It can also be observed from Figure 2 that the intensity of peak (1) is slightly higher than that of peak (2), making peak (2) appear as a shoulder peak of peak (1). Hydration between peak (1) and (2) is controlled by the nucleation process of amorphous AH_3_. Unlike the hydration of OPC and C_3_S [28], there is no dormant period in the hydration process of C_12_A_7_. Moreover, Damidot et al. [9] stated that a short induction period lasting for about 4 h with moderate heat flow existed between peak (1) and (2). A similar phenomenon can also be seen in the work by Edmonds et al. [8]. However, this phenomenon has not been found in our work, even though the hydration heat flow curve is plotted over the same time scale. Reasons may correlate to the properties of sample and experimental technique. Taking the work by Damidot et al. [9] as a case, pure C_12_A_7_ was prepared through the solid reaction between particles of CaCO_3_ and alumina, and the sintering process was followed by a slow cooling period. Therefore, hydration activity of synthesized C_12_A_7_ may be affected. What is more, the whole heat-releasing process during the hydration of C_12_A_7_ starting from the contact between C_12_A_7_ and water has not been well captured in previous work. So far, there is no consensus on the constitution of transition phases. It is well known that C_4_AH_13_ (4CaO·Al_2_O_3_·13H_2_O) would not be generated during the hydration of C_12_A_7_, while the presence of C_2_AH_8_ and CAH_10_ is still under debate [8,9,10,27,29]. Damidot et al. [9,27] proposed a hydration route for C_12_A_7_ wherein C_2_AH_8_ was the unique transition phase, and its formation and conversion processes were described by Equations (5) and (8), respectively. Das et al. [29] claimed that CAH_10_ would be produced as the only transition phase according to Equation (6), and the conversion process followed reaction (9). After systematically investigating the influence of temperature on formation and conversion of transition phases, Edmonds et al. [8] stated that both C_2_AH_8_ and CAH_10_ can be produced during the hydration of C_12_A_7_ at 4 °C, and the formation mechanism was described by Equation (7). However, no trace of CAH_10_ was spotted when C_12_A_7_ hydrated at 20 or 40 °C. The disappearance of CAH_10_ related to the fact that temperature affected the conversion of transition phases greatly. Further, Edmonds et al. [8] pointed out that conversion of C_2_AH_8_ to C_3_AH_6_ (3CaO·Al_2_O_3_·6H_2_O) was significantly accelerated when temperature rose to 40 °C.

(5) $$Ca_{12}Al_{14}O_{33}+51H_{2}O\to 6Ca_{2}Al_{2}O_{5}\cdot 8H_{2}O+2Al{\left(OH\right)}_{3(am.)}\;$$

(6) $$Ca_{12}Al_{14}O_{33}+69H_{2}O\to 6CaAl_{2}O_{4}\cdot 10H_{2}O+2Al{\left(OH\right)}_{3(am.)}+6Ca{\left(OH\right)}_{2}\;$$

(7) $$Ca_{12}Al_{14}O_{33}+60H_{2}O\to 5Ca_{2}Al_{2}O_{5}\cdot 8H_{2}O+2CaAl_{2}O_{4}\cdot 10H_{2}O\;$$

(8) $$3Ca_{2}Al_{2}O_{5}\cdot 8H_{2}O\to 2Ca_{3}Al_{2}O_{6}\cdot 6H_{2}O+2Al{\left(OH\right)}_{3(am.)}+9H_{2}O\;$$

(9) $$3CaAl_{2}O_{4}\cdot 10H_{2}O\to Ca_{3}Al_{2}O_{6}\cdot 6H_{2}O+4Al{\left(OH\right)}_{3(am.)}+18H_{2}O\;$$

(10) $$2CaAl_{2}O_{4}\cdot 10H_{2}O+Ca{\left(OH\right)}_{2}\to Ca_{3}Al_{2}O_{6}\cdot 6H_{2}O+2Al{\left(OH\right)}_{3(am.)}+12H_{2}O\;$$

(11) $$Ca_{12}Al_{14}O_{33}+33H_{2}O\to 4Ca_{3}Al_{2}O_{6}\cdot 6H_{2}O+6Al{\left(OH\right)}_{3(am.)}\;$$

**Figure 3:**
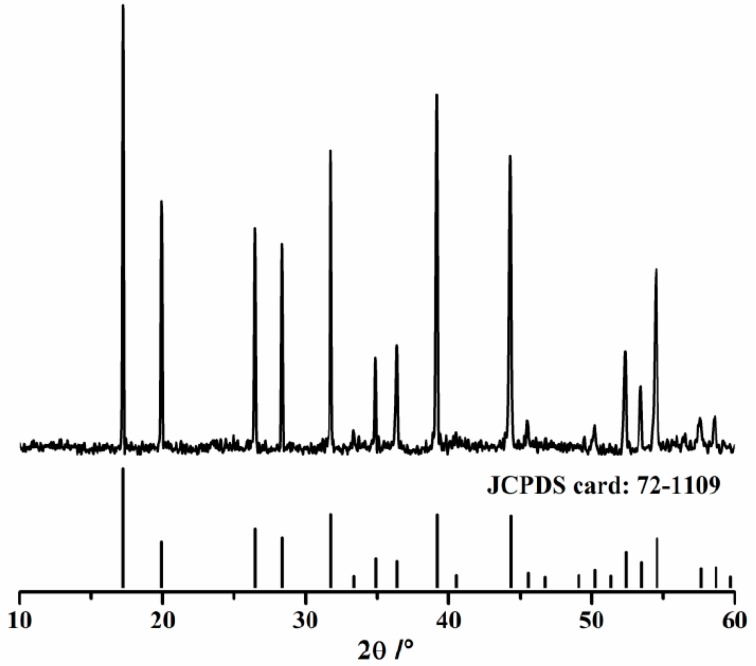
XRD spectrum of hydrated products of C_12_A_7_.

Phase identification of hydrated products of pure C_12_A_7_ is depicted in Figure 3. The sample is gained by hydrating C_12_A_7_ in a sealed bottle for 24 h at 20 °C with a water-to-C_12_A_7_ ratio by weight of 10, and then the suspension is mixed with ethanol to stop hydration. Finally, the mixture is centrifuged and sediments are ground by hand in an agate mortar using acetone as a grinding aid for 10 min, and dried at 50 °C for 7 days in a vacuum oven. As seen in Figure 3, C_3_AH_6_ (JCPDS card: 72-1109) is the only crystal phase in hydrated products and no characteristics of portlandite appear, indicating that Equations (6) and (10) would not happen during the hydration of C_12_A_7_.

Based on the relevant thermodynamic data compiled in Table 3, enthalpy change and Gibbs free energy variance of Equations (5), (7)–(9) and (11) can be calculated. As hydrated products of Equation (6) do not comply to practical founding, enthalpy changes and free energy variances of Equations (6) and (10) are not computed. As seen in Table 4, formation of transition phases is exothermic, whereas the conversion process of these transition phases is endothermic. Therefore, the conversion process of transition phases can be seriously affected by temperature on the micro scale, leading the hydration of C_12_A_7_ to be prominently influenced by temperature on the macro scale.

Based on the above analysis, C_3_AH_6_ and AH_3_ (Al(OH)_3_) may be produced by two ways, i.e., Equation (5)→Equation (8) and Equation (7)→Equation (8) + Equation (9). Their enthalpy change and free energy variance are equivalent to Equation (11), which overall describes the hydration of C_12_A_7_. The coherences manifest that it is difficult to clearly distinguish the reactions incorporated in C_12_A_7_ hydration. However, it can be concluded that C_2_AH_8_ would be definitely generated during the hydration of C_12_A_7_, whereas CAH_10_ is uncertain.

### 3.2. Effect of C_12_A_7_ on Heat-Releasing Behavior

**Figure 4:**
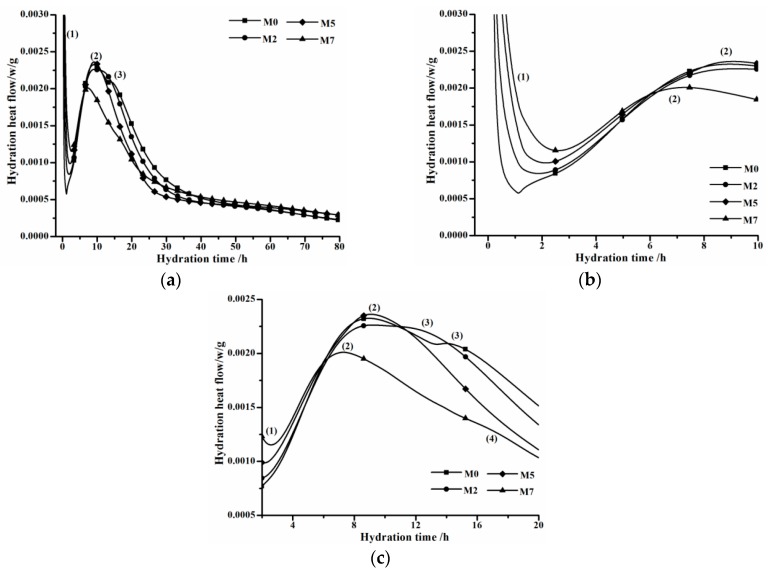
Hydration heat flow curves of samples with various C_12_A_7_ replacements plotted in different hydration time ranges. (**a**) in the rage from 0 to 80 h; (**b**) in the range from 0 to 10 h; (**c**) in the range from 2 to 20 h.

Influence of C_12_A_7_ on hydration of OPC is shown in Figure 4; pertinent samples hydrate at a water-to-complex binder ratio by weight of 0.4, and the sample weights 5 g. Notations of M0, M2, M5 and M7 denote that C_12_A_7_ replacements are 0%, 2.5%, 5.0% and 7.5%, respectively. According to the work by Taylor [15], at most four peaks would appear on the hydration heat flow curve of OPC. In this work, three peaks can be obviously detected during the hydration of each batch. Peak (1) appears immediately when hydration starts, while main peak (2) and shoulder peak (3) emerge at about 8 and 13 h, respectively. However, the last one, peak (4), is less distinct and uneasy to detect. For M7 group, peak (4) occurs at about 16.5 h, while for other groups, peak (4) cannot be observed.

Generally, peak (1) relates to ions dissolving and initial hydration. Main peak (2) correlates to the precipitation of portlandite and C-S-H gel. Shoulder peak (3) is concerned with renewing the formation of ettringite (AFt) [15]. The last and less distinct peak (4) seems to be in connection with the hydration of the ferrite phase or the conversion of AFt to AFm. The rising of peak (1) intensity is caused by the fact that ion dissolving ability of C_12_A_7_ is pretty high, even more rapid than that of C_3_A. Main peak (2) seems independent of C_12_A_7_ replacement, for the regularity between changing tendency of main peak (2) and C_12_A_7_ replacement is not evident. The arrival time of shoulder peak (3) reduces with C_12_A_7_ replacement increasing, and finally it merges with main peak (2). For instance, for M0 and M2 groups, arrival times of shoulder peak (3) are 14.25 and 12.11 h, respectively, whereas for M5 and M7 groups, shoulder peak (3) disappears. The absence of shoulder peak (3) indicates that the hydration of aluminates, especially the formation of ettringite, may be largely affected by C_12_A_7_.

**Figure 5:**
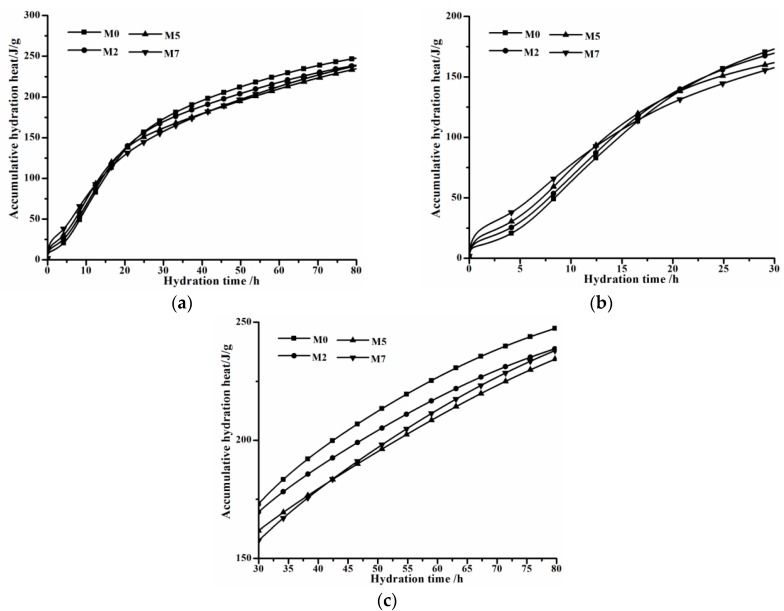
Accumulative hydration heat curves of groups with various C_12_A_7_ replacements. (**a**): in the 0–80 h range; (**b**) in the 0–30 h range; (**c**) in the 30–80 h range.

The influence of C_12_A_7_ on the hydration of OPC can be directly seen from the accumulative hydration heat present in Figure 5. Accumulative hydration heat curves in the whole range are shown in Figure 5a, while two enlarged plots with different hydration time scales are displayed in Figure 5b,c, respectively. It can be observed that C_12_A_7_ promotes initial stage hydration of OPC and then suppresses later hydration. Generally, as C_12_A_7_ replacement rises, accumulative hydration heat before 15 h increases, and then accumulative hydration heat of groups with C_12_A_7_ are all lower than that of control group M0. This phenomenon relates to the fact that a large amount of heat would be released during the hydration of C_12_A_7_, and also hydration activity and ion releasing ability of C_12_A_7_ are pretty high. Compared with the control group, more hydrated products wrap on unhydrated particles in groups with C_12_A_7_, preventing the contact of unhydrated particle from water. Thereby, further hydration of these unhydrated particles is suppressed, resulting in accumulative hydration heats of relevant groups being lower than that of the control group.

### 3.3. Effect of C_12_A_7_ on Hydration Kinetic Processes

**Figure 6:**
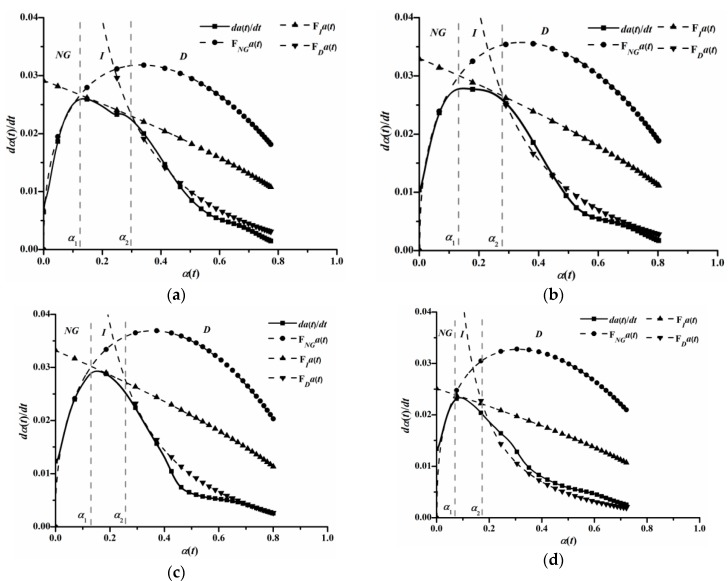
Hydration kinetic process curves of groups with various C_12_A_7_ replacements. (**a**) M0; (**b**) M2; (**c**) M5; (**d**) M7.

The influence of C_12_A_7_ on heat-releasing behavior of OPC on the macro scale may associate with variances of hydration kinetic processes of OPC on the micro scale. Figure 6 shows the analysis results of hydration kinetic processes. Hydration in three stages can be described by Equations (12)–(14), using hydration kinetic parameters such as reaction order and reaction rate constant. As seen in Figure 6, the hydration kinetic model has a moderate fitting effect to M0, M2, M5 and M7 groups.

(12) $$F_{NG}\alpha (t)=k_{NG}\times n\times [1-\alpha (t)]\times {\left[-\ln(1-\alpha (t))\right]}^{\left(n-1\right)/n}\;$$

(13) $$F_{I}\alpha (t)=3k_{I}\times {\left[1-\alpha (t)\right]}^{2/3}\;$$

(14) $$F_{D}\alpha (t)=\left[1.5k_{D}\times {\left[1-\alpha (t)\right]}^{2/3}\right]/\left[1-{\left[1-\alpha (t)\right]}^{1/3}\right]\;$$

Hydration kinetic Parameters are summarized in Table 5. Due to the fact that *NG* stage hydration is highly controlled by autocatalytic reactions of cementitious materials [7], as well as the fact that diffusion resistance of Ca^2+^ in *I* and *D* stages is pretty large, the reaction rate of the *NG* stage is much larger than ones of *I* and *D* stages [33]. It can be observed that both reaction order and reaction rate constant of *NG* stage increase when C_12_A_7_ replacement rises in the range from 0 to 5%, and then reduces with C_12_A_7_ replacement when C_12_A_7_ replacement surpasses 5%. Changing the tendency of reaction rate constants of *I* and *D* stages resembles that of the *NG* stage. However, the hydration kinetic parameter of *D* stage decreases if C_12_A_7_ replacement is higher than 2.5%. It seems that C_12_A_7_ exerts a greater impact on *D* stage than the other two stages.

Hydration activity of C_12_A_7_ is much higher than other clinker phases, such C_3_A, C_3_S, C_4_AF and C_2_S, and also ion releasing ability of C_12_A_7_ is greater than theirs. Correspondingly, Ca^2+^ and AlO_2_^−^ concentrations in pore solution would greatly increase if OPC is substituted by C_12_A_7_, leading to an increase of hydration kinetic parameters of the *NG* and *I* stage. Moreover, the formation rate of hydrated products is also enhanced, this increases the migration barrier for Ca^2+^ diffusion, resulting in a reduction of *I* stage parameters. It can also be observed from Table 5 that all hydration kinetic parameters of the M7 group are lower than the ones of other groups, which is in good agreement with isothermal microcalorimetric experimental results. This phenomenon may concern high C_12_A_7_ replacement, and the mechanism is under further investigation.

The duration of each hydration stage and the corresponding hydration degree are compiled in Table 6. C_12_A_7_ exerts no significant effect on α_1_, while α_2_ consistently decreases with C_12_A_7_ replacement rising. Consequently, the duration of *I* stage steadily reduces with the increase of C_12_A_7_ replacement, suggesting the arrival time of saturation point of Ca^2+^ concentration decreases [32]. Meanwhile, the duration of *D* stage increases with the rising of C_12_A_7_ replacement. Therefore, the hydration kinetic process of OPC has a trend to change from *NG*-*I*-*D* to *NG*-*D*, this coincides well with microcalorimetric experimental results, wherein the arrival time of shoulder peak (3) diminishes with the rising of C_12_A_7_ replacement and finally emerges with main peak (2). As mentioned above, the formation rate of hydrated products is noticeably accelerated after C_12_A_7_ replacing, and more hydrated products would precipitate on the surface of unhydrated particles. Consequently, it becomes more difficult for water to permeate through the hydrated products’ layer, and also the energy barrier of ion migration, such as Ca^2+^, AlO_2_^−^, OH^−^, etc. is higher than before. Hence, the hydration of OPC steps into the diffusion stage early.

### 3.4. Effect of C_12_A_7_ on Hydrate Assemblages

Simulation of hydrate assemblages are performed using the Gibbs free energy minimization program GEMS. According to the work conducted by Lothenbach et al. [34], calculated hydration degrees of individual clinker phases are used as a hydration time-dependent input. Also, the experimental hydration degree of C_12_A_7_ determined in Section 3.2 is used. For the sake of simplicity, interactions among individual mineral phases are not under consideration.

Besides, Fe in the complex binder is predicted to precipitate as amorphous FH_3_ (Fe(OH)_3_) gel, as it is more thermodynamically stable than other phases, like Fe-ettringite, hydrotalcite and carbonates. MgO presented in the cementitious system is predicted to precipitate initially as brucite, and later converts to hydrotalcite. What is more, interactions between sulphates and carbonates are not considered. On one hand, a very tiny amount of thaumasite can precipitate at 20 °C. On the other hand, according to the work by Schmidt et al. [35], thaumasite was only generated under the condition that SO_3_/Al_2_O_3_ molar ratio exceeded 3.0, but in this work, SO_3_/Al_2_O_3_ molar ratio in all samples are lower than critical value. In addition, performance variances of gypsum, hemihydrate and anhydrite are not considered, and are uniformly denoted as gypsum in simulation results.

**Figure 7:**
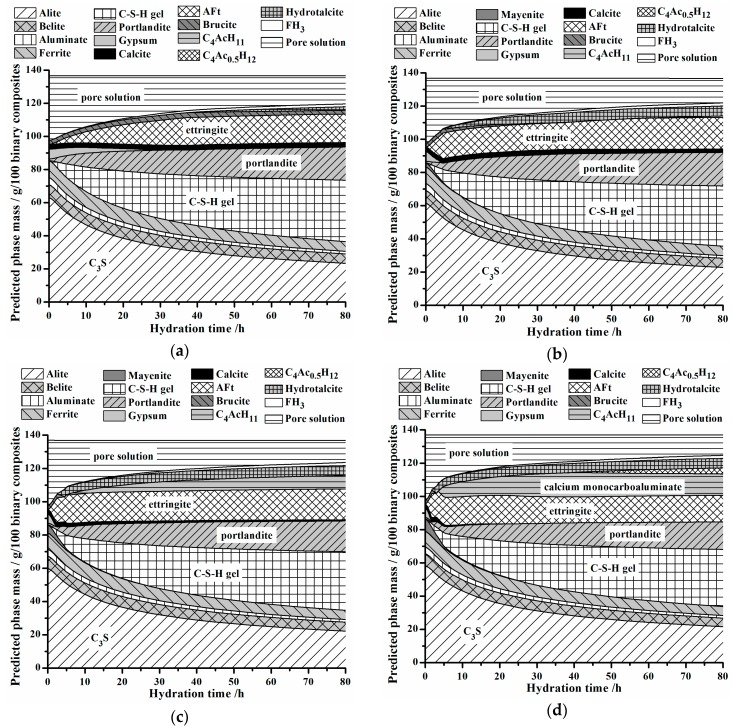
Predicted hydrate assemblages at different hydration ages with w/c = 0.4 at 20 °C. (**a**) M0; (**b**) M2; (**c**) M5; (**d**) M7.

Simulation results of hydrate assemblages are depicted in Figure 7. It can be observed that main hydrated products of OPC are C-S-H gel, portlandite, ettringite, amorphous FH_3_ gel and Mg-containing phases, such as brucite and hydrotalcite, and no trace of C_3_AH_6_ is found. Meanwhile, AFm is absent in all groups. Lothenbach et al. [34] also found an absence of AFm in their studies, and stated that more work should be done in future research.

Basically, hydrate assemblages, consumption rate of gypsum and the role of calcite are affected by the addition of C_12_A_7_. For example, there are no calcium carboaluminates in the M0 group. For the M2 group, calcium monocarboaluminate appears at about 75 h and even calcium hemicarboaluminate presents at ca. 40 h in M7 group. Gypsum is completely consumed at around 1 d for M0 group, while it takes about just 5 h for the M2 group and even shorter for the M7 group.

Generally, simulation results manifest that the formation of carbonate phases like calcium monocarboaluminate and calcium hemicarboaluminate is affected by the addition of C_12_A_7_. Above analysis also makes clear that participation degree of calcite into cement hydration would remarkably increase if OPC is substituted by C_12_A_7_, rather than typically being employed in OPC as an inert filler to mainly reduce the total amount of hydration heat, diminish the potential risk of cracking and raise total economy. Thus, utilization efficiencies of each mineral phase in OPC are profoundly enhanced. 

**Figure 8:**
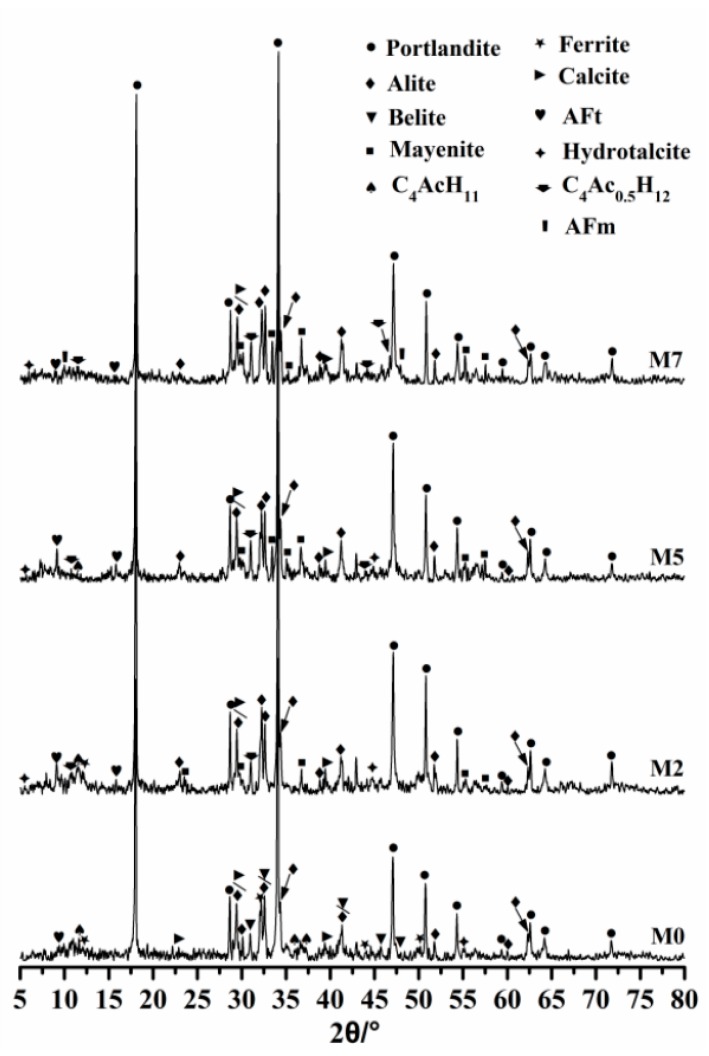
XRD spectra of hydrated products at 3 d age with a w/b = 0.4 at 20 °C.

Simulation results are further verified by the XRD method. Experimental findings of phase identification of hydrated products are shown in Figure 8. These hydrated products are obtained by hydrating related groups at 20 °C for 3 days with a w/b = 0.4. It can be obviously found that dissimilarities occur between experimental results and simulated ones. In practical experiments, C_12_A_7_ still remains even after the sample hydrating for 3 days, and intensities of its diffraction peaks increase with C_12_A_7_ replacement. The presence of C_12_A_7_ is caused by the fact that hydration activity of C_12_A_7_ is considerably higher than that of OPC, and it is difficult to mix C_12_A_7_ and OPC uniformly by hand. Existences of brucite and amorphous FH_3_ gel are unable to be detected by XRD. Moreover, presences of AFt, AFm and hydrotalcite are also uneasy to be observed. For one thing, these materials are in poor crystallinity, and intensities of diffraction peaks are very weak. As seen in Figure 8, intensities of diffraction peaks belonging to hydrotalcite are close to the ones of background noises. Something else to note is that many diffraction peaks of these phases are overlapping with the ones of portlandite, silicates, ferrites and calcite, and thus it is difficult to distinguish their belonging clearly.

Basically, simulation results are in good agreement with the experimental findings. It can be observed in Figure 8 that the intensity of the diffraction peak that appeared at about 2θ = 30.9° increases with C_12_A_7_ replacement, indicating that the amount of calcium hemicarboaluminate rises as well. This coincides with the simulation results very well. Besides, a signal belonging to AFt appears at 2θ = 9.2°, and its intensity increases with C_12_A_7_ replacement except for M7 group. Besides, a less distinct peak that occurs at 2θ = 5.5° signifies the existence of hydrotalcite. Disregarding the amount of each phase, the simulated composition of hydrated products basically conforms to XRD analysis. However, it should be noted that a peak attributed to AFm emerges at 2θ = 9.98°, denoting that AFm is formed in the M7 group. This is inconsistent with the simulation results, meaning the simulation route should be further revised in future investigation.

### 3.5. Effect of C_12_A_7_ on Mechanical Strehgthes

**Figure 9:**
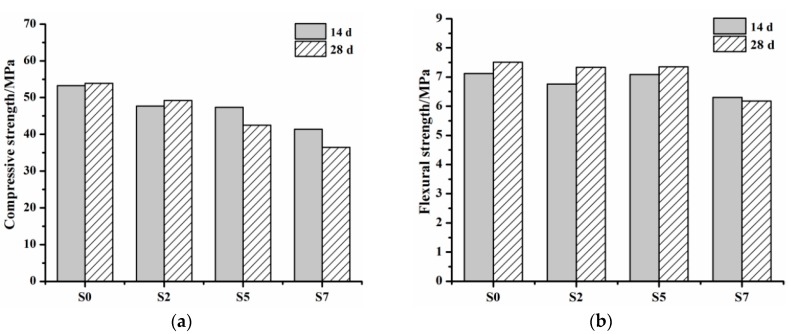
Compressive strength and flexural strength of mortar groups at 14 and 28 d ages. (**a**) Compressive strength of mortar groups at 14 and 28 d ages; (**b**) Flexural strength of mortar groups at 14 and 28 d ages.

The influence of C_12_A_7_ on compressive strength of mortar groups at 14 and 28 d ages is demonstrated in Figure 9a. Basically, the compressive strengths of groups with C_12_A_7_ are lower than that of the reference group, and it can be clearly seen that a reduction of compressive strength with the rising of C_12_A_7_ replacement exists. Moreover, compressive strength shrinkage can be seen in S5 and S7 groups. For instance, compressive strength of S5 group at 28 d age is 10.2% lower than that at 14 d age, and for S7 group, the value is 12.1%. It seems that a higher C_12_A_7_ replacement would produce a larger compressive strength shrinkage. The changing trend of flexural strength of mortar groups at 14 and 28 d ages with the increasing of C_12_A_7_ replacement can be seen in Figure 9b. Unlike compressive strength, flexural strength of groups with C_12_A_7_ stays more or less the same with the reference group if C_12_A_7_ replacement is not higher than 5%. For example, flexural strengths of S0, S2 and S5 groups at 14 d age are 7.1, 6.8 and 7.1 MPa, respectively, and at 28 d age are 7.5, 7.3 and 7.3 MPa, respectively. Similarly, a slightly shrinkage of flexural strength can be seen in the S7 group. For example, flexural strength of the S7 group at 28 d age is 1.9% lower than that at 14 d age. These experimental findings reveal that C_12_A_7_ replacement should be controlled in a reasonable range.

The decrease of compressive strength of mortar groups with C_12_A_7_ comparing to the reference group without C_12_A_7_ may relate to the hydration of C_12_A_7_. Unlike the second hydration behaviors of pozzolanic materials such as fly ash, slag, silica, metakaolin, etc. [36], only the above-mentioned aluminate hydrates would be formed during the hydration of C_12_A_7_. These aluminate hydrates could further react with gypsum or AFt to produce AFm, leading to a reduction of AFt/AFm ratio in hydrate assemblages. Thereby, the skeleton effect of AFt is weakened and microstructure mechanical properties of hydrated products are influenced [37]. Moreover, C_12_A_7_ reacts with water rapidly, and its hydrated products could precipitate on the surface of unhydrated OPC particles, so that the hydration of OPC is affected. This coincides well with the forgoing hydration kinetic analysis results. Besides, rapid hydration of C_12_A_7_ may cause flocculation in the system, and thus defective regions would form in the paste matrix of mortars.

The changing trend of compressive strength with hydration age in this work is a bit different from previous research, wherein compressive strength shrinkage had not been found [13]. This may relate to the fact that a proper amount of other mineral phases such as NaCO, CaSO_4_, NaAlO_2_ and Ca(OH)_2_ were added together with C_12_A_7_ in previous research [13], whereas only pure C_12_A_7_ substitutes OPC in the presented work. The mineral phases may have a combined effect on the properties of hydrated products of OPC, and thus the compressive strength of shotrete could continuously rise at a later age. Since the hydration activity of C_12_A_7_ is pretty high, it is reasonable to postulate that C_12_A_7_ does not possess shape effect, filling effect, second hydration ability, etc., like waste recycled glass [38]. Therefore, the influence of C_12_A_7_ mainly relates to its hydration behavior, properties of hydrated products and interactions of hydrated products between C_12_A_7_ and OPC. In the presented work, the shrinkage of compressive strength perhaps correlates to the conversions among aluminate hydrates in hydrated products of C_12_A_7_.

## 4. Conclusions

Hydration behavior of pure C_12_A_7_ is systematically studied, and its influence on the hydration of OPC is evaluated in the presented work. 

The hydration of C_12_A_7_ involves a transition phase-forming stage and a transition phase-converting stage. Thermodynamic calculation reveals that the transition phase-forming stage is exothermic, while the later converting stage is endothermic. This is why temperature exerts significant effects on the hydration of C_12_A_7_. Metastable transition phase C_2_AH_8_ is produced during the hydration of C_12_A_7_, whereas the formation of CAH_10_ is still uncertain. 

Heat-releasing behavior of OPC can be greatly influenced by C_12_A_7_, and thus accumulative hydration heat increases with C_12_A_7_ replacement rising in the initial 15 h hydration; whereas all accumulative hydration heats of groups with C_12_A_7_ are lower than that of the control group after 15 h.

The influence of C_12_A_7_ on heat-releasing behavior of OPC may be caused by the changing of the hydration kinetic process. All hydration kinetic parameters first rise, and then reduce with C_12_A_7_ replacement increasing. The duration of *I* stage consistently decreases with C_12_A_7_ replacement, showing that the relevant hydration kinetic process has a trend to change from *NG*-*I*-*D* to *NG*-*D*.

Hydrate assemblage of OPC can be greatly affected by C_12_A_7_. The consumption rate of gypsum and calcite can be greatly accelerated by increasing C_12_A_7_ replacement. Participation degree of calcite to hydration would be profoundly enhanced by increasing C_12_A_7_ replacement. Calcium hemicarboaluminate is formed in the high C_12_A_7_ content group. Therefore, the utilization efficiency of calcite typically included in OPC can be adjusted by controlling C_12_A_7_ replacement. It seems that C_12_A_7_ can be employed as a functional component for OPC to adjust the consumption rate of sulphates, and also the amount and type of carbonate phases in hydrated products can be modified.

The influence of C_12_A_7_ on the compressive strength of mortars is greater than on flexural strength. Compressive strength of mortars with C_12_A_7_ is lower than that of the reference group without C_12_A_7_. Meanwhile, compressive strength reduces with C_12_A_7_ replacement increasing. A shrinkage of compressive strength at a later age is detected when C_12_A_7_ replacement is no less than 5%. It seems that the influence of C_12_A_7_ on the compressive strength of mortars mainly relates to its hydration behavior, properties of hydrated products and interactions of hydrated products between C_12_A_7_ and OPC.

In view of the forgoing analysis, C_12_A_7_ has a significant impact on the hydration of OPC. However, interactions among clinker phases are not considered during simulations, and the effects of C_12_A_7_ on their hydration are not taken into account. These issues will be comprehensively researched in future investigations.

## Figures and Tables

**Table 1:** Chemical composition of cement /100 g.

Chemical Composition			Normative Phase Composition		
/g		/mmol	/g		/mmol
CaO	63.525	1132.7	alite	62.9	275.34
SiO_2_	19.25	320.41	belite	7.8	45.37
Al_2_O_3_	3.82	37.47	aluminate	4.4	16.31
Fe_2_O_3_	3.371	21.11	ferrite	10.2	21.09
MgO	2.76	68.49	free-CaO	0.875	15.603
K_2_O	0.746	7.92	CaCO_3_	2.809	28.062
Na_2_O	0.12	1.94	CaSO_4_^d^	5.396	39.636
CO_2_	1.235	28.06	K_2_SO_4_^s^	0.2898	1.66
TiO_2_	0.201	2.52	TiO_2_	0.201	2.517
P_2_O_5_	0.11	0.77	P_2_O_5_	0.11	0.775
SrO	0.207	2.00	SrO	0.207	1.998
MnO	0.103	1.45	MnO	0.103	1.452
SO_3_	3.45	49.09	Na_2_SO_4_^s^	0.3351	0.81
free-CaO	0.875	10.16	K_2_O ^b^	0.157	1.663
readily soluble alkalis ^c^			Na_2_O ^b^	0.0504	0.813
K_2_O	0.589	5.785	MgO ^b^	1.84	45.7
Na_2_O	0.07	0.984	SO_3_^b^	0.078	0.98

**^c^** alkali would dissolve immediately. ^b^ present as solid solution in the major phases of clinker. ^s^ present as alkali sulphates. ^d^ present as anhydrate (0.45 g/100 g), hemihydrate (1.47 g/100 g) and gypsum (3.48 g/100 g).

**Table 2:** Trace elements adopted in main clinker phases [15].

Mineral Phases	Elements /(mmol/100 g)			
	Na	K	Mg	S
Alite	0.48	0.08	27.1	1.06
Belite	0.17	2.9	3.2	0.38
Aluminate	0.52	1.3	4.9	—
Ferrite	0.07	0.4	10.6	—

**Table 3:** Standard thermodynamic properties at 298 K.

Compounds	△H_f_° /(kJ·mol^−1^)	△G_f_° /(kJ·mol^−1^)	S /(J·mol^−1^·K^−1^)	C_p_ /(J·mol^−1^·K^−1^)
Ca_12_Al_14_O_33_ (s)	−19,414.43 ^G^	−18,451.44 ^G^	1044.74 ^G^	1084.83 ^G^
H_2_O (l)	−286 ^M^	−237.2 ^M^	70 ^M^	75 ^M^
Ca_3_Al_2_O_6_·6H_2_O (s)	−5537.25 ^G^	−5008.15 ^G^	421.7 ^G^	445.60 ^G^
Al(OH)_3 (am.)_	−1289 ^L^	−1151.0 ^L^	70 ^L^	36 ^L^
Ca(OH)_2_ (s)	−985 ^L^	−897.01 ^L^	83.4 ^L^	187.51 ^L^
CaAl_2_O_4_·10H_2_O (s)	−5320 ^M^	−4622.4 ^M^	501 ^M^	151 ^M^
Ca_2_Al_2_O_5_·8H_2_O (s)	−5433 ^L^	−4812.8 ^L^	440 ^L^	392 ^L^

**^l^** presents liquid. s means solid. am demonstrates amorphous. ^G^ data is cited from GEMS Nagra-PSI dataset [30]. ^M^ data is cited from Matchei et al. [31]. ^L^ data is cited from Lothenbach et al. [32].

**Table 4:** Thermodynamic parameter changes of reactions might happened at 298 K.

Reaction	Enthalpy /(kJ·mol^−1^)			Gibbs Free Energy/(kJ·mol^−1^)		
	Reactants	Products	Differences	Reactants	Products	Difference
(5)	−34,000.43	−35,176	−1175.57	−30,548.64	−31,178.8	−630.16
(7)	−36,574.43	−37,805	−1230.57	−32,683.44	−3308.8	−625.36
(8)	−16,299	−16,226.5	72.5	−14,438.4	−14,453.1	−14.7
(9)	−15,960	−15,841.25	118.75	−13,867.2	−13,881.75	−14.55
(11)	−28,852.43	−29,883	−1030.57	−26,279.04	−26,938.6	−659.56

**Table 5:** Parameters determined by hydration kinetic model.

Sample	*NG*			*I*		*D*	
	*n*	K*_NG_*/(μm^2^·h^−1^)	*sd*	K*_I_*/(μm^2^·h^−1^)	*sd*	K*_D_*/(μm^2^·h^−1^)	*sd*
M0	1.67523	0.040978	0.00237	0.009704	0.00528	0.002187	0.00326
M2	1.715074	0.045435	0.0027	0.010967	0.00484	0.002263	0.00191
M5	1.792231	0.046102	0.00252	0.011083	0.00457	0.002136	0.00238
M7	1.591035	0.043168	0.00186	0.008372	0.00367	0.001025	0.002

*sd* denotes standard deviation.

**Table 6:** Critical points of hydration degree and duration of each hydration stage.

Sample	Hydration Degree		Duration of Each Hydration Stage		
	α_1_	α_2_	*NG*	*I*	*D*
M0	0.1258	0.2997	0.1258	0.1739	0.7003
M2	0.1328	0.2775	0.1328	0.1447	0.7225
M5	0.1296	0.2617	0.1296	0.1321	0.7383
M7	0.0750	0.1680	0.0750	0.0930	0.8320

α_1_ and α_2_ represent hydration degrees. Duration of *NG* stage equates to the value of α_1._ Duration of *I* stage calculated by subtracting α_2_ from α_1_. The rest hydration period belonging to the duration of *NG* stage determined as 1-α_2_.

## References

[1] Mohamed B.M., Sharp J.H. (2002). Kinetics and mechanism of formation of tricalcium aluminate, Ca_3_Al_2_O_6_. Thermochim. Acta.

[2] Nishio Y., Nomura K., Miyakawa M., Hayashi K., Yanagi H., Kamiya T., Hosono H. (2008). Fabrication and transport properties of 12CaO·7Al_2_O_3_ (C_12_A_7_) electride nanowire. Phys. Status Solidi A Appl. Res..

[3] Tong Z.F., Xie S.L., Zhang L.H., Chen T., Liu W. (2011). Preparation and application of the 12CaO·7Al_2_O_3_ material. Nonferr. Met. Sci. Eng..

[4] Hirao H., Yokoyama S. (2003). Behavior of chloride ions in hardened eco-cement: A new type portland cement made from municipal waste incinerator ash. Concr. Res. Technol..

[5] Xie Y.G., Sheng X., Wang Y.C., Li L.H. (2008). Study on sulfate treatment in water by mayenite. China Water Waste Water.

[6] Wang Q., Yang J.W., Yan P.Y. (2012). Influence of initial alkalinity on the hydration of steel slag. Sci. China Technol. Sci..

[7] Han F.H., Wang D.M., Yan P.Y. (2014). Hydration kinetics of composite binder containing different content of slag or fly ash. J. China Ceram. Soc..

[8] Edmonds R.N., Majumdar A.J. (1988). The hydration of 12CaO.7Al_2_O_3_ at different temperatures. Cem. Concr. Res..

[9] Damidot D., Rettel A. Effect of gypsum on CA and C_12_A_7_ Hydration at room temperature. Proceedings of the 11th International Congress on the Chemistry of Cement (ICCC).

[10] Park C.K. (1998). Characteristics and hydration of C_12−*x*_A_7_·*x*(CaF_2_) (*x* = 0~1.5) minerals. Cem. Concr. Res..

[11] Park H.G., Sung S.K., Park C.G., Won J.P. (2008). Influence of a C_12_A_7_ mineral-based accelerator on the strength and durability of shotcrete. Cem. Concr. Res..

[12] Won J.P., Hwang U.J., Kim C.K., Lee S.J. (2013). Mechanical performance of shotcrete made with a high-strength cement-based mineral accelerator. Constr. Build. Mater..

[13] Won J.P., Hwang U.J., Lee S.J. (2015). Enhanced long-term strength and durability of shotcrete with high-strength C_12_A_7_ mineral-based accelerator. Cem. Concr. Res..

[14] Rovnanik P. (2011). Influence of C_12_A_7_ admixture on setting properties of fly ash geopolymer. Ceram. Silikaty.

[15] Taylor H.F.W. (1997). Portland cement and its major constituent phase. Cement Chemistry.

[16] Castel E., Ik Shin T., Fourcade S., Decourt R., Maglione M., García-Víllora E., Shimamura K. (2012). Effect of annealing under O_2_ and H_2_ on the piezoelectric parameters of the Ca_12_Al_14_O_33_ single crystals. J. Appl. Phys..

[17] GB/T 17671-1999 (2014). The Quality and Technology Supervision Bureau. Method of Testing Cements—Determination of Strength.

[18] GB/T 175-2007 (2015). The Quality and Technology Supervision Bureau. Common Portland Cement.

[19] Dong J.H., Li Z.Y. (2010). Effect of Temperature on Heat Release Behavior of Hydration of Cement. J. Build. Mat..

[20] Hewlett P.C. (2004). The constitution and specification of portland cement. Lea's Chemistry of Cement and Concrete.

[21] Krstulović R., Dabić P. (2000). A conceptual model of the cement hydration process. Cem. Concr. Res..

[22] Yan P., Zheng F. (2006). Kinetics model for the hydration mechanism of cementitious materials. J. Ceram. Soc..

[23] He Z., Yang H., Hu S., Liu M. (2013). Hydration mechanism of silica fume-sulphoaluminate cement. J. Wuhan Univer. Technol. Mater. Sci. Ed..

[24] He Z., Yang H.M., Liu M.Y. (2014). Hydration Mechanism of Sulphoaluminate Cement. J. Wuhan Univer. Technol. Mater. Sci. Ed..

[25] Zhou H., Liu J., Liu J., Li C. (2012). Hydration kinetics process of low alkalinity sulphoaluminate cement and its thermodynamical properties. Procedia Eng..

[26] Tian Y., Jin X.Y., Jin N.G. (2010). Micro expressions of cement hydration kinetics. Rare Met. Mater. Eng..

[27] Damidot D., Sorrentino F. Modification of the hydration process from C_12_A_7_ to C_3_A at 20 °C. Proceedings of the 10th International Congress on the Chemistry of Cement (ICCC).

[28] Kirby D.M., Biernacki J.J. (2012). The Effect of water-to-cement ratio on the hydration kinetics of tricalcium silicate cements: Testing the two-step hydration hypothesis. Cem. Concr. Res..

[29] Das S.K., Mitra A., Das P.P.K. (1996). Thermal analysis of hydrated calcium aluminates. J. Therm. Anal. Calorim..

[30] Thoenen T., Kulik D. Nagra/PSI chemical thermodynamic database 01/01 for the GEM-Selektor (V.2-PSI) Geochemical Modeling Code, PSI. http://les.web.psi.ch/Software/GEMS-PSI/doc/pdf/TM-44-03-04-web.pdf.2003..

[31] Matschei T., Lothenbach B., Glasser F.P. (1997). Thermodynamic properties of portland cement hydrates in the system CaO-Al_2_O_3_-SiO_2_-CaSO_4_-CaCO_3_-H_2_O. Cem. Concr. Res..

[32] Lothenbach B., Matschei T., Möschner G., Glasser F.P. (2008). Thermodynamic modelling of the effect of temperature on the hydration and porosity of portland cement. Cem. Concr. Res..

[33] Han F.H., Zhang Z.Q., Yan P.Y. (2012). Early hydration properties of steel slag under high alkalinity. J. Chin. Electron Micros. Soc..

[34] Lothenbach B., Winnefeld F. (2006). Thermodynamic modelling of the hydration of portland cement. Cem. Concr. Res..

[35] Schmidt T., Lothenbach B., Romer M., Scrivener K. (2008). A thermodynamic and experimental study of the conditions of thaumasite formation. Cem. Concr. Res..

[36] Paul S.C., van Rooyen A.S., van Zijl G.P. (2018). Properties of cement-based composites using nanoparticles: A comprehensive review. Constr. Build. Mater..

[37] He Z., Cao G.X., Li Y., Liu L.L. (2016). Influence of potassium-sodium ratio on the early age cracking sensitivity of cementitious materials with high alkalinity. J. Hydraul. Eng..

[38] Paul S.C., Šavija B., Babafemi A.J. (2018). A comprehensive review on mechanical and durability properties of cement-based materials containing waste recycle glass. J. Clean. Prod..

